# Assessing Routing Strategies for Cognitive Radio Sensor Networks

**DOI:** 10.3390/s131013005

**Published:** 2013-09-26

**Authors:** Suleiman Zubair, Norsheila Fisal, Yakubu S. Baguda, Kashif Saleem

**Affiliations:** 1 UTM-MIMOS Centre of Excellence in Telecommunication Technology, Faculty of Electrical Engineering, Universiti Teknologi Malaysia, 81310 UTM Johor Bahru, Malaysia; E-Mails: sheila@fke.utm.my (N.F.); baguda_pg@fke.utm.my (Y.S.B.); 2 Center of Excellence in Information Assurance (CoEIA), King Saud University, 11195 Riyadh, Saudi Arabia; E-Mail: ksaleem@ksu.edu.sa

**Keywords:** *ad-hoc* networks, cognitive radio, cross-layer, wireless sensor network, routing

## Abstract

Interest in the cognitive radio sensor network (CRSN) paradigm has gradually grown among researchers. This concept seeks to fuse the benefits of dynamic spectrum access into the sensor network, making it a potential player in the next generation (NextGen) network, which is characterized by ubiquity. Notwithstanding its massive potential, little research activity has been dedicated to the network layer. By contrast, we find recent research trends focusing on the physical layer, the link layer and the transport layers. The fact that the cross-layer approach is imperative, due to the resource-constrained nature of CRSNs, can make the design of unique solutions non-trivial in this respect. This paper seeks to explore possible design opportunities with wireless sensor networks (WSNs), cognitive radio *ad-hoc* networks (CRAHNs) and cross-layer considerations for implementing viable CRSN routing solutions. Additionally, a detailed performance evaluation of WSN routing strategies in a cognitive radio environment is performed to expose research gaps. With this work, we intend to lay a foundation for developing CRSN routing solutions and to establish a basis for future work in this area.

## 1. Introduction

The need for efficient spectrum utilization [1] has recently brought about the new paradigm of cognitive radio sensor networks (CRSNs). The two major drives toward this paradigm are the underutilization of the spectrum below 3 GHz and the congestion problem in both licensed and unlicensed bands. As challenging as this paradigm may appear, the effort of recent studies such as [2,3] are gradually making this paradigm a reality.

Meanwhile, as the World gradually develops into an Internet of Things, the ubiquity of wireless sensor networks (WSNs) is accordingly becoming imperative. This, by implication, further complicates the issue of the congestion of the industrial, scientific and medical (ISM) spectrum and the unlicensed national information infrastructure (UNII), as evidenced by [4–6]. Notwithstanding the predicted ubiquity of WSNs, other wireless systems such as WiMAX, Bluetooth and Wi-Fi also operate in these bands, along with cordless phones and microwaves. The normal IEEE 802.15.4 standard defines 16 channels, each with a bandwidth of 2 MHz, in the 2.4-GHz ISM band, among which only four are not overlapping with the IEEE 802.11 22-MHz bandwidth channels. It should be noted that these channels sometimes overlap with the channels of IEEE 802.11. If the Wi-Fi deployment uses channels other than 1, 6 and 11, then overlapping will occur. Furthermore, a recent and practical study performed on the co-existence issue showed that, in reality, only three of these channels are actually non-overlapping [7]. In extreme cases where all other networks (e.g., medical sensor networks, security networks, disaster communications, PDAs, Bluetooth devices and many more applications envisioned in the very near future) compete for these four channels, the congestion issue becomes more urgent. The authors of [8,9] have shown that IEEE 802.11 degrades the performance of 802.15.4 when they operate in overlapping bands, and in [7] a highly variable IEEE 804.15.4 performance drop of approximately 41% was demonstrated. Furthermore, as computing/networking heads toward ubiquity, various WSNs will form a great percentage of this phenomenon. The concept of CRSN aims to address this spectrum utilization challenge by offering sensor nodes temporary usage of vacant primary user (PU) spectra via dynamic spectrum access (DSA) with the condition that they will vacate that spectrum once the presence of the incumbent is detected.

With the successful implementation of DSA via cognitive radio (CR), other advantages are exploited by the WSN. The most enticing of these advantages are that the node energy can be significantly conserved by the reduction of collisions, which invariably results in the reduction of retransmission of lost packets. Energy conservation can also be achieved by employing nodes that dynamically change their transmission parameters to suit channel characteristics, thus providing full management control of these valuable resources. This practice, in effect, can also enable the coexistence of various WSNs deployed in a spatially overlapping area in terms of communication and resource utilization.

Notwithstanding the potential of this concept, the CRSN comes with its own unique challenges. For example, the practical development/implementation of a CR sensor node is still an unsolved issue. Additionally, because the DSA characteristic affects the entire communication framework of a conventional WSN [2], previous protocols proposed for classical WSNs cannot be directly applied to a CRSN, nor can the communication protocols for *ad-hoc* networks perfectly fit this context due to the resource constraints. Incorporating the idea of DSA into a WSN changes not only the MAC and PHY layers, but also affects all of the communication. However, the fact that WSNs still remain the launch pad for protocol design in CRSNs necessitates a performance study of WSN routing strategies *vis-à-vis* CRSN requirements [2,10,11]. Thus, there is a need for specially adapted communication protocols to fulfill the needs of both DSA and WSNs in a CR context.

The network layer is fundamental in any network and is significantly affected by the dynamic radio environment created by CR because it addresses the peer-to-peer delivery through other nodes in a multi-hop fashion to the correct recipients in due time. The sending node must address both its dynamic radio environment and that of the next hop node. This phenomenon is otherwise referred to as the “deafness problem” and introduces a challenging scenario requiring innovative algorithms that consider the intrinsic nature of the sensor nodes. This scenario necessitates a cross-layer approach for designing spectrum-aware routing protocols. A number of researchers have proposed routing schemes for cognitive radio *ad-hoc* networks [12]. However, due to the differences in constraints between classical ad-hoc networks and WSNs, these solutions cannot be directly imported to solve the problem of routing in CRSNs. Although CRSNs can also be *ad-hoc* in nature, they differ from classical *ad-hoc* networks in the following ways:

- Sensor networks (SNs) are usually densely deployed, with hundreds of nodes, because the harsh atmosphere to which the nodes are exposed can easily cause node failures. In contrast, *ad-hoc* networks are not usually densely deployed.
- While SNs are highly constrained with respect to memory, energy and computation capabilities, *ad-hoc* networks usually do not consider these fundamental constraints.
- The mode of communication in a SN is usually based on broadcast, whereas *ad-hoc* networks use point-to-point mode most of the time.
- SNs usually have the communication goal of data aggregation, in addition to the plain communication goal of *ad-hoc* networks.
- Addressing schemes in SNs are significantly different from those applied in traditional *ad-hoc* networks because of the enormous overhead of schemes such as IP addresses and GPS coordinates.
- Finally, SNs have periods in which they “sleep” to conserve energy, whereas nodes in most *ad-hoc* networks do not have this property.

To the best of our knowledge, specific attention has not been given to routing in the network layer of CRSNs, although recent research has emphasized the transport [10,11], MAC and physical layers [10,12,13]. Hence, there is the need for research to focus on this area. We present a review of WSN routing strategies *vis-à-vis* CRSN requirements to evaluate the strengths and weaknesses of each strategy. This review is provided to enable protocol designers to use quantitative evidence in selecting the strategies best suited to their application. The paper then discusses the factors affecting routing CRSNs, reviews recent studies in this area and categorizes them appropriately. Open issues in this respect are also identified. The paper further identifies major CRSN routing components and presents a systematic review of relevant studies in each category to reveal the open issues.

The main contributions of this paper are as follows:

- To identify a research gap in the network layer of CRSNs.
- To evaluate WSN routing strategies *vis-à-vis* CRSN requirements.
- To propose cross-layer and routing frameworks for routing in CRSNs.
- To discuss the main components of routing in CRSNs *vis-à-vis* recent studies to reveal open areas.

The rest of this paper is organized as follows: Section 2 provides a general overview of CRSNs, defining the main building blocks of the field. Recent research trends in this respect are also mentioned. Section 3 examines routing in WSNs, presents issues arising from the introduction of the CR component and discusses the cross-layer design concept. Section 4 presents a performance evaluation of WSN routing strategies with respect to DSA. This paper seeks to be a pioneer in this regard. Section 5 discusses the results of the simulations described in Section 4. Section 6 discusses state-of-the-art routing in CRSNs and describes open research areas. Section 7 discusses routing issues in CRAHNs *vis-à-vis* CRSN requirements. Section 8 presents CRSN routing preferences and a routing framework. Section 9 is dedicated to routing in CRSNs *vis-à-vis* current studies in this field while mentioning open areas of research. Section 10 concludes the article.

## 2. Overview of Cognitive Radio Sensor Networks

This section presents a brief overview of CRSNs, which is a paradigm built upon WSNs, by identifying its main features and summarizing recent research trends.

### 2.1. Wireless Sensor Networks (WSNs)

WSNs are traditionally characterized by sensor nodes deployed in an *ad-hoc* (self-organizing) manner with communication and resource constraints and a fixed spectrum allocation. Based on the implemented topology, the sensors can communicate with each other directly or indirectly through routers. Each node has sensing, processing and communication capabilities. The nodes can serve as both data sources and routers. Based on these features, the node can possess other functionalities [14,15].

### 2.2. What is a CRSN

As defined by [2], a CRSN is a distributed network of wireless cognitive radio sensor nodes that sense an event signal and collaboratively communicate their readings dynamically over the available spectrum bands in a multi-hop manner to satisfy application-specific requirements.

#### 2.2.1. Main Features of a CRSN

For a CRSN to gain any rational meaning, it must adopt the intrinsic characteristics of WSNs while still performing CR functions. Thus, a WSN is expected to benefit from the features of CR, such as DSA and the power consumption reduction achieved by adaptability. The nature of throughput is expected to be bursty due to opportunistic channel usage, which mitigates the problem of an increased probability of collision in densely deployed WSN environments. In the past, because of the low throughput of traditional WSNs, congestion and over-flooding were not significant design issues. However, with the bursty nature of throughput in CRSNs, these issues must be addressed, especially in real-time applications that consider quality of service (QoS).

One of the pioneering studies in this field [16] clearly demonstrated how CRSNs outperform traditional WSNs based on a comparative protocol study between a standard ZigBee/802.15.4 sensor and a CR-based version of the same sensor. The results showed the superiority of CRSNs over WSNs based on hop count, throughput and end-to-end application layer latency, without incurring significant overhead.

#### 2.2.2. Recent Research Trends in CRSNs

Different cognitive approaches other than CR have been advanced for WSNs [17,18]. When considering CRSNs, most of the research trends have followed DSA approaches that are usually restricted to the MAC [16]. Exceptions are [10,11], which analyzed the effect of employing common WSN transport layer protocols in a CRSN environment. This gap demonstrates the need for research to develop effective CRSN routing protocols.

## 3. Routing

This section presents the major issues that arise in CRSN routing, after discussing the nature of routing in WSNs. The concept of incorporating cross-layering is also discussed *vis-à-vis* routing in CRAHNs. Finally, we present a proposed routing framework for CRSNs based on the reviewed studies.

### 3.1. Routing in WSN

Generally, protocols in WSNs are application-based. Hence, universal communication protocols do not exist because all applications consider varying factors that influence their design. Routing in a WSN is not similar to routing in other networks because most WSNs are data centric and require the flow of data from many sources to a sink. Hence, due to the intrinsic nature of the network, combined with its unique constraints and self-organizing nature, multi-hopping is employed to send data to the sink. Based on the underlying network structure, WSN routing protocols can generally be classified as flat, hierarchical, location-based or QoS-based. Ref [13,19] have attempted to provide details about the available protocols in this respect.

### 3.2. Routing in CRSN: Rising Issues

Although the inefficiency of traditional WSN routing strategies has been theoretically discussed, there has not been an analytical evaluation of the performance of these strategies in a CRSN environment to expose the need to include DSA capabilities in existing WSN routing algorithms. As explained above, CRSNs with the capability of opportunistic utilization of both licensed and unlicensed bands are introduced to combat the spectrum scarcity and congestion issues in the case of dense deployment, which is a major characteristic of sensor networks. The DSA component imposes unique challenges to routing in WSNs, which are outlined as follows.

#### 3.2.1. Control Signaling

Efficient control signaling is critical to any routing protocol, and its implementation depends on the routing approach from source to sink. In a traditional WSN, control signaling design is not an important issue because the channel is usually pre-assigned before field deployment. However, in the case of CRSNs, the dynamic nature of the available channel makes the task of designing a control channel quite challenging. By implication, CRSNs incur more overhead than WSNs in terms of energy and communication to negotiate a control channel. Efficient schemes in this regard should be characterized by minimizing this additional overhead. Again, most applicable algorithms designed for traditional ad-hoc networks evidently do not consider minimizing this overhead as an issue because there is an abundance of resources. Because CRSN topology can be either *ad-hoc*, clustered, hierarchical or mobile, it should also be noted that topology dictates the most effective algorithm to adopt. Below are the three design methods:

(a). Dedicated common control channel (DCCC): In the DCCC method, a dedicated channel in the ISM band is usually assumed to be available across the width of the network and is strictly assigned to all nodes for signaling. This idea is very simple and can easily support any of the four topologies of a CRSN. Notwithstanding, the practicability of this idea has always been doubtful, especially for large networks. Another setback of this scheme is its high susceptibility to entire network failure from a simple jamming attack to the DCCC.
(b). Group-based control channel: In this case, a control channel is assigned by a cluster head in a strict cluster association or in a distributed manner [20] in virtual clusters. The strength of this scheme lies in its support for spatial frequency reuse in large networks. However, in the case of strict cluster association, channels are assigned randomly or in a dedicated fashion to separate clusters based on the decision of a fusion center, which is usually assumed not to be constrained in any capacity. This assignment makes the former more suited to data mining applications. In the case of virtual clusters, a control channel is decided upon in a distributed manner, which makes it a better option for both *ad-hoc* and clustered topologies and real-time applications.
(c). Sequence-based control channel negotiation: This scheme takes inspiration from Bluetooth communication in that unsynchronized nodes run channel-hopping algorithms until the nodes meet at the same channel, after which they exchange synchronization packets and hop over a sequence for data exchange. In this case, the efficiency of an algorithm depends on the time required for nodes to meet at the control channel. The energy expended on communication in this scheme is greater than that of the two methods described above. Again, the algorithm is bested suited to peer-to-peer communication and will be highly demanding in terms of latency when extended to multi-hop communication.

#### 3.2.2. Spectrum Sensing/Licensed User Interference

Spectrum sensing introduces silent periods in the nodes and hence reduces the time required for a duty-cycled node to attempt a data transfer. This reduction arises because nodes cannot perform spectrum sensing and data transfer at the same time. Thus, reducing the sensing period increases the probability of interfering with primary user activity and also increases channel access duration. However, based on the licensed user activity and the route management algorithm, the sensing period can be varied. The other option is to use multiple front-ends for dedicated activities. This option, in addition to its cost implications, compromises the simplicity of the nodes.

#### 3.2.3. Opportunistic Spectrum Access (OSA)

OSA capabilities allow a node to maximize the utilization of channel user activity. CRSN nodes must be aware of the nature of primary user activity in such channels to efficiently perform OSA. The consequences of being oblivious to the user's activity, as in the case of traditional WSN routing that lacks OSA, include more packet drops, frequent communication black-out periods and heightened latency that arise when the protocols must wait for a transmission.

#### 3.2.4. Spectrum Decision/Coordination

The spectrum decision of a node greatly affects the process of routing and can indefinitely initiate route rediscovery processes if not properly coordinated. Hence, routing nodes must coordinate their spectrum decision, especially for real-time applications.

#### 3.2.5. Intermittent Connectivity

Upon the arrival of the primary user, the sensor node must vacate the current channel and perform spectrum handoff. This process introduces added delays and has the potential of increasing loss rates in the network because the route to the sink is constantly changing or interrupted.

#### 3.2.6. Cross-Layering Approach

The success of designing efficient protocols for CRSNs lies in adopting the cross-layer approach. Even in the realms of WSNs, some solutions have adopted this approach for routing [21,22] to improve the routing performance. Thus, because of the benefits of adopting this design approach, more researchers are adopting it as a practice in various areas. However, the cross-layer approach usually adopted in these schemes [21,22] involves a one-way directional flow of information due to the traditional layered communication approach that is usually employed in protocol design.

**Figure 1.**
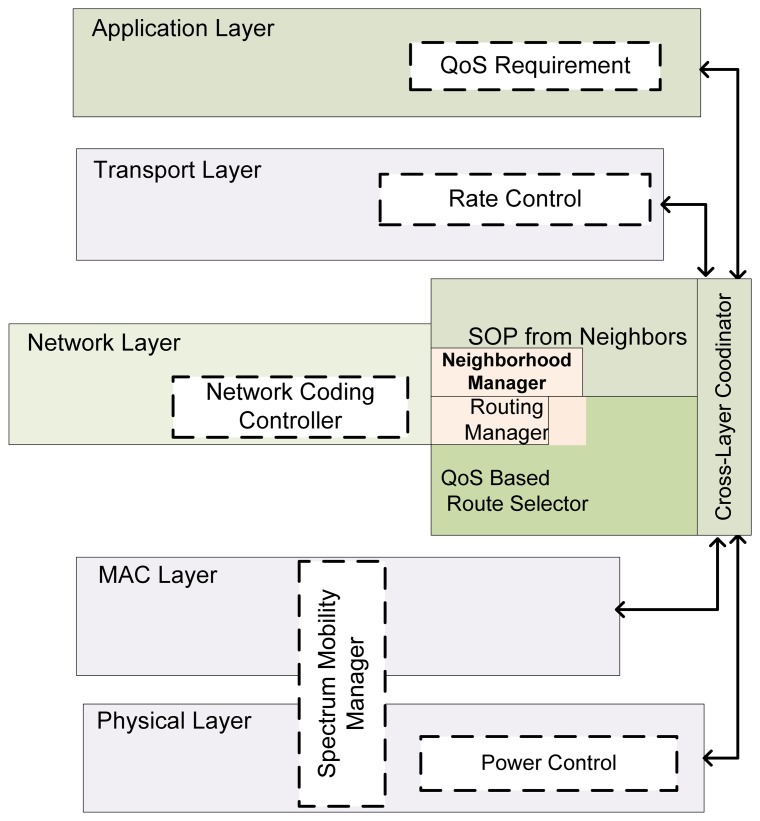
Cross-layer framework communications.

However, in reality, the interrelationship/interdependency of the layers in the CRSNs is bi-directional. As shown in Figure 1, both the spectrum mobility and management functions affect all communication layers. For example, the latency introduced by spectrum handoff adversely affects both the routing protocols and the transport layer protocols.

Additionally, the application layer can stipulate its channel condition requirements or can request a spectrum handoff, just as the physical and MAC layers can also state their available conditions to the application layer, and adjust appropriately. Likewise, [23] demonstrated the interdependency between local contention (MAC layer) and end-to-end congestion (transport layer). Finally, routing must fully consider spectrum handoff and medium contention (MAC) alongside the action of routing [23] to meet specific QoS demands. The manner in which variations in channel properties affect routing has also been explored by [21,22].

The concept of cross-layering in the design of protocols does not seek to change the traditional information flow in the established layered communication model. Rather, all information needed from each layer (depending on the design) is usually stored in a dedicated buffer for individual layers to access before processing any data in their domain. Thus, the standard communication paradigm still holds. This concept of cross-layering alongside the CR at the physical layer actually adds cognition to the entire layer. This effect will produce better network performance in all scenarios.

#### 3.2.7. Cross-Layer Framework

Although this concept has been widely utilized, there is no generalized framework for cross-layer interactions that shows the limitations and opportunities. Figure 1 presents a cross-layer framework in the context of CRSNs that can easily be generalized to other areas.

Depending on the task at hand, a QoS route selector/controller (QRC) is proposed to form the bidirectional link between the required layers to achieve optimum cross-layering. The operation can be explained as the QRC obtaining the application QoS demand from the application layer. This result arises because various QoS demands expect minimum requirements to be met regarding channel characteristics, node buffer state and latency. However, some of these requirements can be traded off for others to cushion the limitation of one of the factors. In this regard, differentiated service can easily be characterized. Thus, based on the spectrum opportunities (SOP) available to the QRC, the MAC layer can perfectly allocate channels that appropriately suit the various local demands by carrying out a rule-based contention. This idea can prove very effective in multimedia networks.

It is expected that nodes that are qualified to take part in routing will send the SOP and piggyback other information such as the buffer state, sending rate, *etc.*, depending on the routing protocol being deployed. Furthermore, based on the SOP of neighboring nodes, network coding opportunities can easily be identified and utilized when network coding is implemented on the network. Although some researchers have proposed adding a separate coding plane to the communication layers [24], this method perfectly solves the case of network coding-aware schemes. Such information is used for both power and sending rate control in the physical layer.

A mobility manager that handles spectrum sensing, spectrum sharing and spectrum handoff will also need information from the QRC to efficiently perform these responsibilities. This module is hosted by the network layer with the network coding module. The QRC is expected to perform optimization based on its needs and requirements. Determining the most appropriate scheme, which can prove to be a non-trivial task, is left to the discretion of the designer based on the purpose of the design.

To justify the need to consider the various factors presented in this model, we must evaluate the performance of WSN routing strategies with respect to DSA.

## 4. Evaluating the Performance of WSN Routing Strategies with Respect to DSA

**Figure 2.**
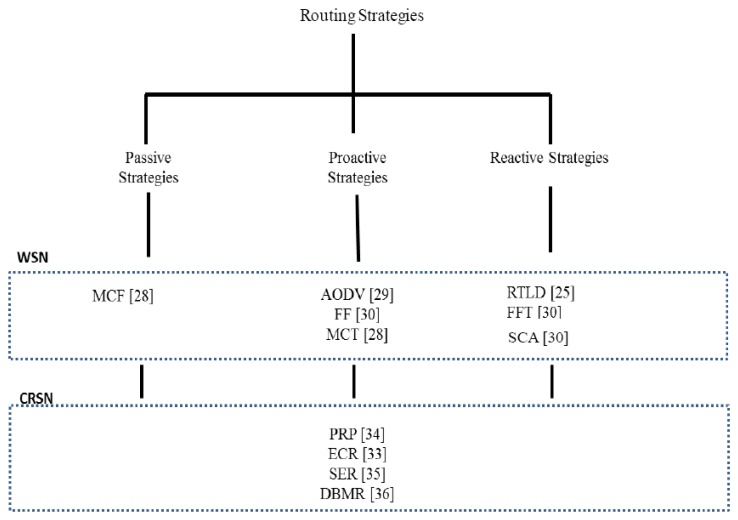
Classification of routing protocols with respect to transmission strategy.

DSA is the major capability that CR introduces to WSNs. DSA addresses how nodes access the media in an opportunistic manner (*i.e.*, opportunistic spectrum access—OSA). Media access in WSNs can either be contentious (CSMA) or time-based (TDMA). WSN routing strategies with respect to the technique of medium access can be categorized into passive, active and proactive, which all lack OSA capabilities, as shown in Figure 2. Thus, their performance varies when implemented in a CRSN environment without integrated OSA. We conduct a detailed performance evaluation of these strategies to further expose the need to incorporate OSA into WSN routing.

### 4.1. Passive Strategy

Such routing protocols do not consider the link state of the receiving node while sending data towards the sink. Data packets are usually flooded in all available paths to the sink in a constrained manner or without any constraint at all. A good representation of this strategy is message-initiated constrained flooding (MCF) routing [25]. While flooding-based strategies generally decide whether to broadcast at each hop, in MCF flooding, the decision is regulated by a cost function known as the Q-value, indicating the minimum cost to the destination from each node, which can be found if not known *a priori*. This value is updated every time a node receives a packet from its neighbors. Two techniques are used to control the flood: (a) the transmit time difference is appended to the broadcast so that nodes with better estimates transmit first while suppressing duplicates or (b) if the receiving nodes estimate a higher cost than the transmitting node, the node only updates its cost and refrains from broadcasting.

### 4.2. Proactive Strategy

Protocols in this category first secure a path (or the best path) to the sink before routing data packets along the chosen route. Examined protocols that adopt this strategy include the *ad-hoc* on-demand distance vector (AODV) routing protocol [26], flooded forward ant routing (FF) [27] and QoS-aware learning-based adaptive spanning tree meta-strategy routing (MCT) [25].

#### 4.2.1. Ad-hoc on-Demand Distance Vector (AODV) Routing Protocol

When a node has data to send to the sink and its neighborhood table entry is either outdated or has no source to the sink, it generates and broadcasts a route request (RREQ) packet, which is forwarded using various metrics (according to the modified method employed) to the sink. At each hop, every node searches its neighborhood table for a valid route to the sink and will only rebroadcast the RREQ when none is found. The node then sets a backward pointer to the neighbor from which it received the RREQ packet. When the RREQ packet reaches the sink, the sink will generate a route reply (RREP) packet and will unicast it along the found path to the source; then, the data transfer will begin. If the source receives more than one RREP packet, it selects the final route based on the minimal hop count or the best link quality. In the advent of a route failure during data transfer, a route error (RERR) packet is generated and is unicast back to the node from which the message initiated. This packet flushes the corresponding routing table entries in the intermediate nodes. The version implemented for this evaluation employs cross-layer techniques to avoid paths characterized by high packet losses.

#### 4.2.2. Flooded Forward Ant Routing (FF)

In FF, forward ants are flooded to search for the destination, and backward ants that help guide the ants back to the source are created by the forward ants that find the sink. During the backward ant stage, the cost between each hop and the link probabilities are updated. Multiple paths are updated by one flooding phase. Flooding is stopped if the probability distribution is good enough for the data to reach the destination. The rate of release for the flooding ants is reduced when a shorter path is traversed. Two strategies are used to control the forward flooding. First, the distance to the sink is evaluated by the link probability *P_n_*=1/|N| (where n represents an ant's neighbor and N is the set of neighbors), and this distance is used to determine which neighbor will first broadcast a forward ant to join the forward search. Second, a random delay is added to each transmission such that another node that overhears the same ant from other neighbors will drop its own copy of the ant.

#### 4.2.3. QoS-Aware Learning-Based Adaptive Spanning Tree Meta-Strategy Routing (MCT)

In MCT, an adaptive spanning tree is constructed based on a meta-strategic reinforcement learning module. The forwarding phase passes received packets to a node's parent. All nodes that overhear this transfer will update their corresponding NQ-value (the communication cost to the corresponding node) and re-estimate their own Q-value (the minimum cost to the sink from this node) as shown in Equation (1). If the packet is not received by the forwarded node within a certain period, the NQ-value is updated to be the largest among the neighbors and the parent pointer is reset to the neighbor with the minimum cost:

(1) $$Q_{m}=\left(1-\alpha \right)Q_{m}+\alpha \left(o_{m}+\underset{n}{\min}NQ_{m}\left(n\right)\right)$$

where *α* is the learning rate, *o*_m_ is the current value of the local objective function, and *n* is the corresponding neighbor of the node.

### 4.3. Reactive Strategy

In the reactive strategy, instead of securing a path before data transmission, the path search is conducted in an online fashion alongside the data transfer. Examples of this include the real-time load distribution routing protocol (RTLD) [22], flooded piggyback ant routing (FFT) [27] and scan ant routing (SCA) [27].

#### 4.3.1. Real-Time Load Distribution Routing Protocol (RTLD)

In RTLD, nodes always maintain an updated neighborhood table that records the values of the velocity to route packets to all neighbors, the packet reception rate and the remaining power. This information is gathered by means of a periodic broadcast of “hello” packets to help refresh the neighborhood table. Routing is then performed based on the geographical location of the nodes in a quadrant. Nodes that have data packets to send select only eligible recipients that reside in its quadrant until the packet is routed to the sink. Hence, the best path to the sink is explored based on the three gathered metrics from the “hello” broadcast.

#### 4.3.2. Flooded Piggyback Ant Routing (FFT)

In FP, the forward search ant is combined with the data ant, and the control of both is based on two strategies. First, the distance to the sink, evaluated by the link probability *P_n_*=1/|N| (where n represents an ant's neighbor and N is the set of neighbors), is used to determine which neighbor will first broadcast a piggybacked ant. Second, a random delay is added to each transmission such that other nodes that overhear the same piggybacked ant from another neighbor drop their own copy of the data. The probability distribution constrains the flooding towards the destination for future data ants.

#### 4.3.3. Scan Ant (SCA)

Just as in RTLD, each node broadcasts a forward ant to gather the probability distribution of all of its neighbors Q_n_, where n is a neighbor. Afterwards, the cost to the destination is calculated based on Equation (2):

(2) $$C=\min_{n\in N}\left(c_{n}+Q_{n}\right)$$

where c_n_ is the local cost function. The initial probability is calculated according to Equation (3):

(3) $$p_{n}\leftarrow \frac{e^{{\left(C-Q_{n}\right)}^{\beta }}}{\sum_{n\in N^{e(C-Q_{n})\beta }}}$$

### 4.4. Simulation Setup

The simulation was performed using RMASE in Prowler [28]. RMASE [29] is an application implemented in the probabilistic wireless network simulator Prowler, which provides realistic and simple radio/MAC models. RMASE supports a layered routing architecture for plug-and-play common routing components. This application consists of a network topology model, an application model and a performance model. RMASE was developed for dealing with the challenge of comparing different routing algorithms for sensor networks. Both the physical layer and the medium access layers were modified to include licensed user activity, which is modeled to be independent. This simulation uses a simple signal interference noise ratio (SINR) radio propagation model based on [28] and is represented by:

(4) $$P_{\text{rec},\text{ideal}}\;(d)\leftarrow P_{\text{transmit}}/\;\left(1+d^{\gamma }\right)$$

(5) $${\text{P}}_{\text{rec}}\;(\text{i},\text{j})\leftarrow {\text{P}}_{\text{rec},\text{ideal}}\;({\text{d}}_{\text{i},\text{j}})\cdot (1+\alpha (\text{i},\text{j}))$$

(6) $${\text{P}}_{\text{rec},\text{pu}}\;(\text{d})\leftarrow {\text{P}}_{\text{rec},\text{ideal}}\;(\text{d})\cdot (1+\alpha (\text{d}))$$

In Equation (4), *P_transmit_* represents the sender's signal strength, and *P_rec,ideal_(d)* is the ideal received signal strength at a distance d between two nodes *i* and *j*. The fading model parameter α is a random variable with a normal distribution *N(0,σ_α_)*. According to this model, a signal can only be received if the SINR limit as perceived from both neighbors and the primary users is above a threshold Δ*_n_ and* Δ*_p_*, respectively, along with a value of receiver noise variance (*RNV*). By default, the only transceiver is tuned to the data channel. The presence of interference from the neighboring nodes or the primary user is determined by comparing the received power in Equation (5) with Δ*_n_* and in Equation (6) with the sensing thresholds Δ*_p_*. Thus, the inter-arrival of the licensed user is modeled based on an exponential distribution. As a two-state, ON-OFF process with ON rate β and OFF rate α, the state transition follows a Poisson arrival process. Based on this assumption and using renewal theory, posteriori probabilities of both states are calculated as follows:

(7) $$P_{ON}=\frac{\beta }{\beta +\alpha };P_{\text{OFF}}=\frac{\alpha }{\alpha +\beta }$$

Each sensor node is attached to a single transceiver, and the medium access control layer used has carrier-sense multiple access with collision avoidance (CSMA/CA). Table 1 shows the relevant RMASE simulation parameters. After setup, each scenario was run five times, and an average was taken.

### 4.5. Performance Metrics

The considered performance metrics are as defined below.

(1). Latency: Measures the time (in seconds) it takes to send a message from source to sink. This term is a function of the number of hops, the packet transverse length of transmission queues, random MAC delays and routing delays (which are based on the strategy a protocol uses to avoid collision).
(2). Throughput: Measures the time performance for the entire network. This term is defined as the number of messages the sink receives per second (Kbits/s).
(3). Success Rate: Measures the overall success of the network as a percentage (%). This term is computed as the total number of packets received at all of the destinations versus the total number of packets sent from all of the sources.
(4). Loss Rate: The number of lost packets against the total expected number of packets for the sink, measured as a percentage (%). This term measures the quality of the protocol.
(5). Energy Consumption: The sum of used energy (in Joules) of all of the nodes in the network for transmitting, receiving, spectrum sensing and idling.

Some of the simulation parameters presented in Table 1 require some explanation. Flood Temp is a temperature variable used to control the flood. The higher the value of T, the higher is the chance that a packet is broadcasted. The MCBR learning rate is described in Equation (1). Resend represents the number of times the node resends a packet in the instance of loss. ForwardDelta sets the forwarding status of all nodes. MaxDelay is the maximum delay granted to a packet before it is declared as a timeout (simulation equivalent of 1 s, *i.e.*, 40,000 = 1 s, this conversion is the same for all scenarios).

AntStart is the time that elapses before the search ant is sent. Ratio is the ratio of control packets to data sent. RewardScale is a simulation parameter used to scale the reward function. DataGain is an initialization parameter used to calculate the total number of packets sent. Window Size is the memory reserved for queue management. The C1 and Z initialization simulation values are used to compute the probability of initial search ants being broadcasted.

## 5. Comparing Strategies with Protocols

**Figure 3.**
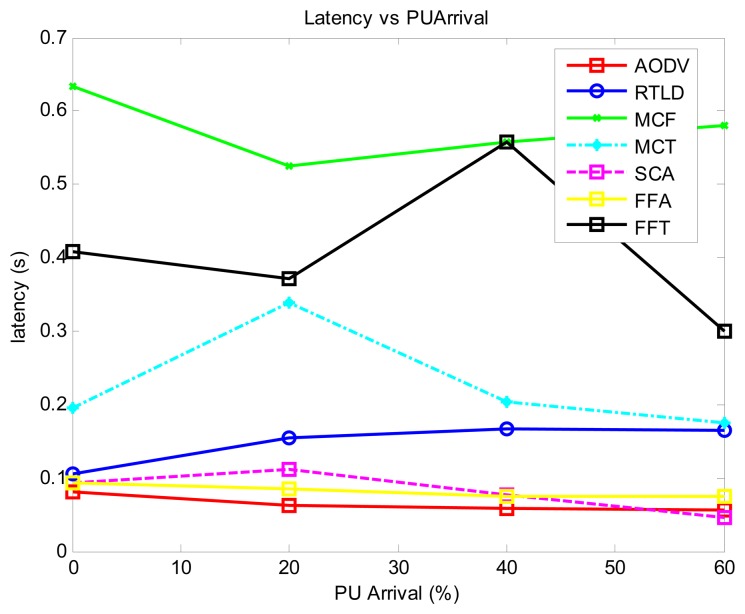
Latency performance of all protocols *versus* primary user activity.

As illustrated in Figure 3, we find that the proactive strategies AODV and FFA generally record the best performance in latency because the path search is performed offline and is separated from the data transmission phase. The MCT performance is lower because the hierarchical tree is only formed for the nodes to be aware of the sink location. However, the data routing phase is still performed in a contentious manner among neighboring nodes, which initiates more back-off in the process of data routing. The passive strategy (MCF) records the highest latency because much of the flooding nature of the algorithm makes channel access contention a significant factor in addition to the PU activity. The reactive strategies record moderate latency performance, with SCA recording the best, even comparable to the reactive strategies. This performance is achieved because the reinforcement learning module quickly adjusts to the PU environment in an online mode.

**Figure 4.**
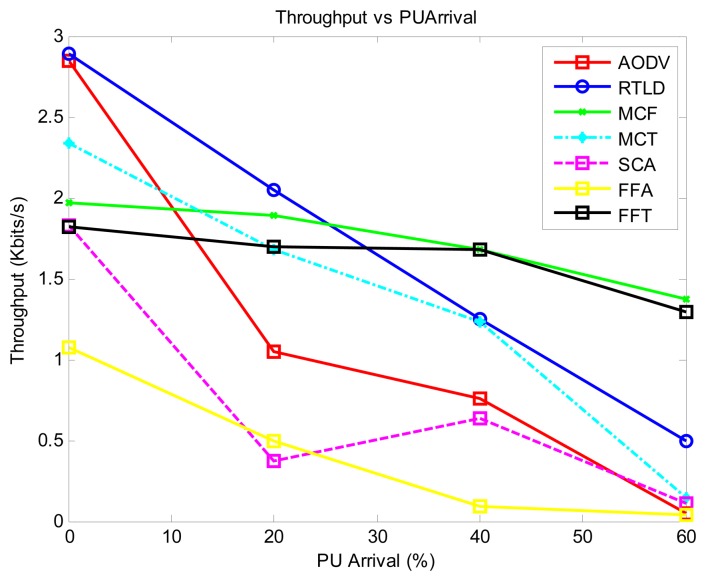
Throughput performance of all protocols *versus* primary user activity.

Generally, as shown in Figure 4, the throughput decreases with increasing PU activity. The passive strategy (MCF) exhibits the best performance in this regard due to the number of data duplicates injected into the network. The proactive strategies MCT and AODV display a moderate performance, with FFA having the lowest throughput. In general, the online strategies RTLD and FFT exhibit the best stable time performance. FFA has the worst throughput in this regard because the time required to service the route failures impacts the throughput.

**Figure 5.**
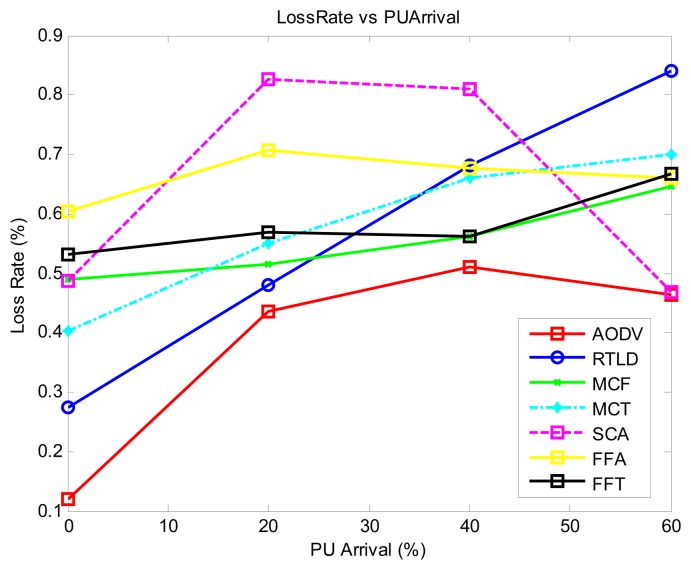
Loss rate of all protocols *versus* primary user activity.

With regard to the quality of the protocol (Figure 5) with medium PU activity, most of the protocols display stable performance of the loss rate, with SCA showing the highest loss rate. However, this loss is drastically reduced at a high PU activity of 40%–60% because of its online reinforcement learning ant strategy. This result is contrary to the general trend of increasing loss rate with increasing PU activity exhibited by all of the protocols. Overall, the proactive AODV protocol shows the lowest loss rate. RTLD contends with increased online activity as PU activity increases, increasing the loss rate.

**Figure 6.**
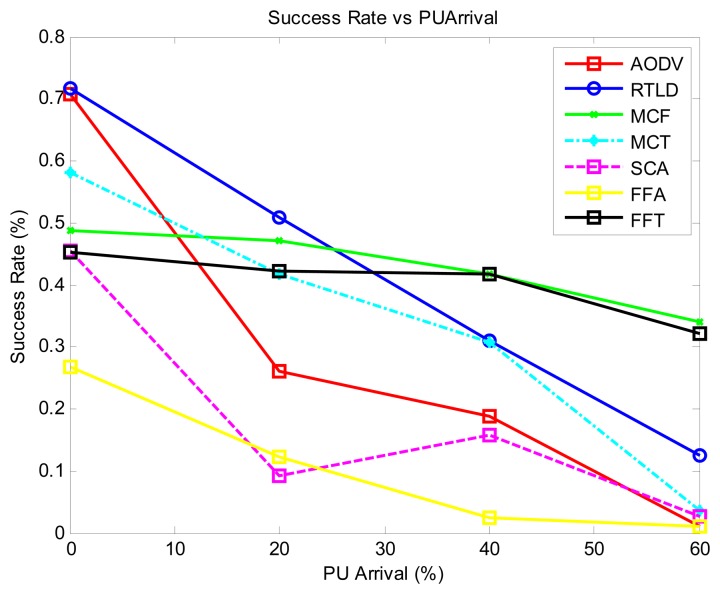
Success rate of all protocols *versus* primary user activity.

In Figure 6, the protocols that achieve general network success in such dynamic networks are those that inject more duplicate data: MCF and FFT. However, the AODV strategy still performs better than others. This approach is comparable to MCF and FFT, with the advantage of reducing duplicate packets in the network.

**Figure 7.**
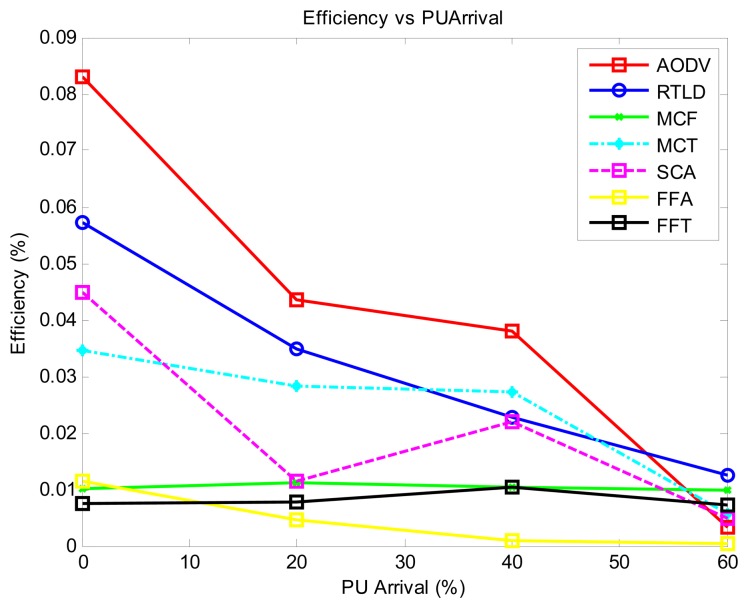
Energy efficiency of all protocols *versus* primary user activity.

The proactive strategies AODV and MCT generally exhibit the best energy efficiency (Figure 7). The poor performance of MCF, FFT and FFA is due to the increased data processing, whereas online decisions and activity are responsible for the poor performance of SCA.

## 6. Recent Studies on CRSN Routing

Recent relevant studies have consistently chosen the proactive approach to the design of routing protocols for CRSNs because this strategy provides an easy way to manage the dynamic nature of the topology by securing a path to the sink before beginning the data transfer stage. However, this strategy falls short in online opportunistic utilization of the dynamic topology, due to the varying status of the channels. Thus, the final route decision cannot be justified as the optimal route at any time. Hence, proactive strategies manage the situation rather than taking advantage of it. Additionally, because of this property, the proactive strategy can experience increased delays during the data transmission stage, except when alternative techniques are incorporated at the cost of complexity and energy overhead. The protocols in this respect are described below.

It is important to note that routing in CR-based networks must generally be considered with the spectrum sensing decision [30,31]. Thus, protocols relevant to routing in CRSNs can generally be categorized as shown below.

### 6.1. Joint Route and Spectrum Brokering

In this method, the choices of spectrum and path are made jointly by the individual routing nodes or by the sink node after a path is chosen. Thus, the chosen route remains connected during the routing process. Energy- and cognitive-radio-aware routing (ECR) [32], which is analogous to the AODV protocol, is based on this concept. In ECR, the RREQ packet is broadcasted to the sink through a common control channel. Intermediate nodes forward the RREQ based on channel correlation with the sending node, energy threshold and channel availability. When multiple routes are found, the sink chooses the route with the least number of hops and further assigns the operating channel to individual nodes to reduce channel switching during the data transfer phase. Route maintenance is only performed locally if the affected node is in close proximity to the sink. Otherwise, a message must be sent back to the source to initiate a new route request, which can be costly. Another problem with this approach is that the channel availability metric is not properly accounted for.

Another protocol under this classification is spectrum and energy-aware routing (SER) [33]. Although SER is not specific to CRSNs, the protocol takes energy into consideration and thus can be classified under viable solutions for CRSNs. The protocol also presents another modification of the AODV protocol and differs from the others based on its distributed joint routing and channel-timeslot allocation strategy for each link. However, CSMA/CA is still used at each link for channel access. The protocol excels in network balancing of energy consumption, reduction of contention in the MAC between nodes and the ability to decompose traffic over different channels or timeslots. However, a detailed implementation method of the MAC component is lacking, which leaves the assumptions open for verification. Furthermore, the ability of the constrained nodes to maintain periodic spectrum sensing to obtain an informed channel occupancy characteristic on which they base their decision is a critical matter of concern.

### 6.2. Reconfigurable Joint Route and Spectrum Brokering

This category of routing protocols is characterized by the ability to recover from changes in the spectrum caused by PU arrival. The probabilistic routing protocol based on priori information (PRP) [34] expands upon the Dijkstra routing algorithm by introducing a routing metric that enables the nodes to select channels and routes based on the documented performance of the channels during previous transmissions. The metric is formulated based on naïve Bayes inference and uses an m-estimate probability to make the route decisions more realistic. The source node first broadcasts a route discovery packet. This packet is disseminated across all of the nodes to the sink. This packet enables individual nodes to calculate the cost function of choosing any of its neighbors based on the formulated routing metric. At this stage, channels are tagged to neighbor nodes. During the process of routing, when the PU arrives, the affected node can easily change path without jeopardizing the entire routing process. Notwithstanding the ability of the network to reconfigure the route, the energy required to implement the Dijkstra routing algorithm can prove to be non-trivial.

### 6.3. Joint Route and Spectrum Brokering with PU Awareness

Basically, routes in any CR network must provide a measure of protection for the ongoing communication of the PUs. This protection can be implemented either by avoiding the regions known to have high PU activity entirely or by jointly allocating transmission power to incur a greater number of hops and to minimize the probability of interfering with the primary receivers. Protection can also be implemented using underlay or overlay cognitive radio technologies.

In an underlay system, secondary users are allowed to share the channel simultaneously with primary users. Underlay systems protect primary users by enforcing a transmission power constraint on the secondary signals so that the interference generated by the secondary devices is below an acceptable noise floor for the primary users of the spectrum. The interference power constraints associated with underlay systems allow only short-range communication, which can be suitable for CRSNs. Overlay systems, on the other hand, allow concurrent transmissions of primary and secondary users with the condition that the secondary users can use part of their power for secondary communication and the remainder of the power to assist (relay) primary transmissions. Thus, a careful choice of the power split can precisely offset the decrease in the primary user's SNR due to the interference caused by the secondary transmission with the increase in a primary user's SNR due to the assistance from secondary relaying [35,36].

Thus, it can be concluded that underlay CR can only be used when there is a strong primary signal, whereas overlay CR is more appropriate for weak primary signals.

In line with this approach, distributed best-route selection for multipath routing (DBMR) [37] investigates multipath routing selection in performance optimization under energy-constrained CRAHNs via distributed and heuristic routes selected by secondary users (SUs) with the aim to improve end-to-end delay while taking energy into consideration. Multipath routing is modeled as a restless bandit stochastic process optimization problem that allows secondary users to select routes considering the dynamic occupancy of a licensed spectrum and energy based on a finite-state Markov chain (FSMC) model. To protect the PU transmission, the SU's transmission power in each hop varies with the PU's occupancy in each channel. Again, as noted in SER [33], the energy required for frequent spectrum sensing in addition to the increased energy demand of maintaining multipath capability still remains a critical issue.

Other related works in this regard include [38], which only incorporates the channel selection metric into the cluster head and does not introduce a routing strategy. [39,40] propose a routing method for cognitive sensor networks based on a high capacity cluster head or gateways, which is inconsistent with ideal scenarios. In contrast, [41] details the process of collecting relevant routing parameters, such as estimates of neighboring PUs and SUs and channel usage statistics, without providing a clear routing framework.

### 6.4. Open Issues

Based on the above discussion, it becomes clear that there is a need for extensive research efforts to address the discussed challenges and to develop effective network layer solutions along the open issues outlined below.

- There is an urgent need for research efforts to incorporate reactive energy-efficient data transfer components into proactive strategy-based protocols. This effort should be aimed at replacing the usual route repair via the broadcast of route requests used in recent studies.
- Because constant periodic spectral monitoring is nearly impossible for constrained nodes, there is a need to study analytical frameworks that implement energy-aware, cooperative, opportunistic spectrum access for the optimization of routing.
- There is a need for the development of adaptive schemes that consider various QoS requirements for application-specific purposes.
- An optimal method of employing multipath or flooding methods must be investigated for data mining applications.

## 7. Routing in CRAHNs

Unlike the dedicated spectrum communication scenario (WSN), route establishment in CRSNs is more challenging and more complex due to the dynamic nature of spectrum opportunities at each CR node at specific times. The source's spectrum opportunities might be completely different from those available at the sink. Thus, a secured route for data transmission can move across various channels in various spectra as it correlates with the next hop spectrum opportunities. For exchanging spectrum opportunities among neighboring nodes, either the synchronization window or the common control channel (CCC) approach is usually implemented.

Based on the generalized routing framework for CR networks presented by [42], in contrast to the classical routing tables of ad-hoc networks that hold only the next hop information, the routing table is usually extended in CRAHNs to contain the full channel information and the characteristics of the next hop. Channel switching decisions are made with respect to the information received from the QoS evaluation block, the learning block and the information obtained from the sensing activity. The learning block systematically arranges the next hop channel history based on previous QoS demands via learning algorithms. Based on this framework, it is expected that the network will gradually become fully aware of its environment to dramatically reduce or eliminate the on-communication delays caused by switching activity.

When taking CR into consideration, routing protocols in classical ad-hoc networks can generally be classified as follows: (1) A spectrum with next hop selection-based protocols; in this case, the routing metric taken into consideration is simply the spectrum allocation along the selected route; (2) Spectrum selection with PU awareness/avoidance-based protocols; here, other metrics considered with spectrum allocation include PU location and power adaptation; (3) Spectrum selection with reconfigurable route-based protocols that consider spectrum switching and route recovery as well as spectrum allocation.

Moving toward designing effective solutions in this regard, [12,43] extensively review the challenges and directions based on spectrum knowledge categorization. Below, we present some recent studies in this area, including the following.

The CRP [44] is a distributed routing protocol built upon the AODV scheme in terms of route discovery. The CRP supports multiple classes of routes based on the application's QoS requirement. Its unique property lies in its spectrum selection mapping metrics and its local PU awareness to delay forwarding packets. Relevant metrics properly depicting these values can be carefully chosen and tuned to fit the protocol requirements. Its use of the CCC favorably reduces computational overhead. The percentage utilization of the CCC has been shown to be approximately 15%–22%, which implies that the control and channel coordination traffic is rather infrequent. The CRP is operated in two phases. First is the spectrum selection phase during which the chosen route is service-classified into two class I routes, whose optimization functions seek to maximize the propagation distance and the longest allowed time for transmission. The class II route, in contrast, gives precedence to complete PU avoidance even at the expense of latency and especially for the PU receivers that are generally not detected. The second stage is the net-hop selection phase, when each node maps its route participation preference to a forwarding delay and the final route is chosen based on the arrival time of the RREQ requests. As is typical with ad-hoc networks, neither computational complexity nor energy was considered in any of its functions; thus, it is completely unsuitable for the sensor network realm.

Another solution that also offers service differentiation based on traffic priorities is OSDRP [45], which is implemented for mesh networks. However, in this case, the priority for the end-to-end traffic flow is given to routes that offer minimum delay and maximum stability, while also considering the latency introduced by the spectrum handoff. The algorithm is based on four modules: route discovery, route decision, transmit power control (ORTPC) and route maintenance. The route discovery module discovers all possible paths between the source and the sink node by a neighborhood exchange of spectrum opportunities via RREQ packets. The module arranges its discovery according to various latency values offered by each potential route. The route decision module choses the latency value from a sorted list to best fit the requirement of the traffic at hand. ORTPC then adjusts the transmit power according to the traffic requirement. The route maintenance module is responsible for ensuring that all entries in the routing table are updated. Although the transmit power varies in this scheme, the continuous exchange of spectrum opportunities among neighboring nodes and the continuous updating of routing entries can be very taxing on the energy-constrained nodes in a WSN.

Other schemes consider the full knowledge of the network topology with respect to what they offer before making the routing decision. For example, [46] uses the elaboration of algebraic connectivity metrics to capture network connectivity, network diameter and average node distance. Then, weights are assigned to paths according to PU interruption. The gymkhana routing scheme then makes the final route decision after collecting all of the possible route information. However, having full knowledge of the network topology in a WSN is virtually impossible because of the associated cost and the density of node deployment. In summary, routing in CRNs can be categorized into routing with spectrum decision, routing with joint spectrum decision and PU awareness and routing with joint spectrum decision and re-configurability.

## 8. Routing Preferences in CRSNs

Although [47] clearly illustrated how the joint route outperforms the discrete route both with spectrum selection in CRSNs, routing in a CRSN comes with its unique challenges:

- On-demand routing is favored over predetermined routing [48] because the latter is not suitable for the dynamic network scenario created by DSA.
- The hop count is reduced to prolong network life, which results in new metrics to be considered in the process of designing the routing protocol, such as bandwidth, channel access delay, interference and operating frequency.
- Re-routing is caused by the spectrum handoff.

**Figure 8.**
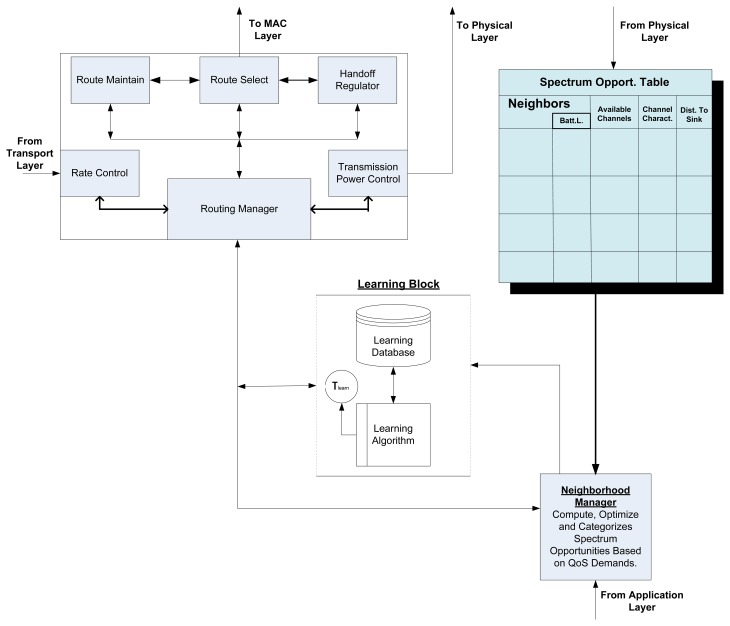
CRSN routing framework.

From the review above, we come to the conclusion that in any practical solution for routing problems in a CRSN, various issues must be considered. We have attempted to summarily capture these issues in the form of a summarized framework, as shown in Figure 8. Although the issues are not completely generalized, the framework summarily shows the design issues that must be considered.

Central to this model is the routing manager, which is the main building block and regulates route selection control, route maintenance control, rate control and power control. The routing table is fed with spectrum opportunities of the node and its neighbors. The node battery capacity, the characteristics of each available link and the buffer status of the node are also recorded. Depending on the scheme being implemented, the routing table can be further reduced by setting the criteria for any node that will participate in the transmission. The spectral opportunities are computed and categorized by the neighborhood manager, and only relevant spectrum opportunities that meet the criteria from the application layer are sent after the selection process is optimized *vis-à-vis* the QoS stipulations. The criteria can be simply formulated into classes of differentiated services that can be categorized based on the delay and link reliability offered by one set of links against that offered by the others. These classes can be summarized into soft and hard real-time services according to the level required by the network services. Thus, links can be tagged to the various offered services. The learning block is fully aware of all decisions made at specific times for specific conditions of specific services and systematically stores the decisions. The block gradually gains cognition about its environment such that, with time, it makes the necessary decisions without having to engage in a vigorous optimization computation to save energy. Depending on the dynamics of the spectrum environment experienced by the node, it might choose to increase or reduce its learning time space (τ_learn_). This aspect will, by implication, have a great impact on reducing the quiet periods usually introduced by the sensing activity and will thus give the node more time for data transmission. Hence, this component can bring about enhanced throughput.

Based on the available information fed to the route manager, it selects a route, manages the route and opportunistically regulates the power of transmission. The performance of each link in terms of transmission interruption due to PU arrival is fed back to the learning block to update the route priority list. Depending on the handoff scheme, whenever a handoff is initiated from a particular link due to PU arrival, it is stored in the memory with other layer information (as explained in Section 3.2.7) readily available to the network layer. All of these instructions are encapsulated in the header and are attached to the packet payload before it is sent to the lower layers.

## 9. Routing in CRSNs

In this section, we discuss the basic modules that compose routing in CRSNs and their related solutions and identify specific research gaps that need to be filled.

### 9.1. CRSN Routing Modules

Again, routing protocols in CRSNs are application-specific, *i.e.*, there exist no strict standardized protocols for all applications. Thus, depending on the application, protocols are accordingly designed to efficiently suit the specific purpose. However, any routing protocol for a CRSN must consider the major issues of network topology, route setup and route management.

#### 9.1.1. Network Topology Management

**Figure 9.**
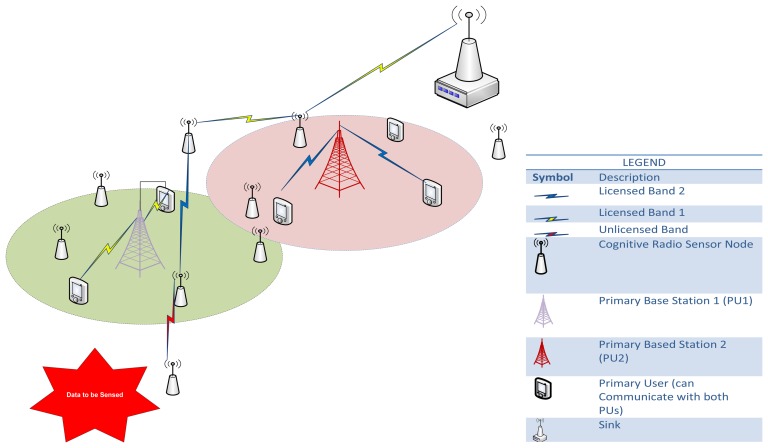
Illustration of a typical routing scenario in a CRSN.

CRSNs usually exhibit network topologies that best suit their application. These topologies can be ad hoc, clustered, hierarchical or mobile in nature. However, the ever-changing spectrum opportunities of the nodes give CRSNs a dynamic topology because a node can seize the availability of a vacant low-frequency band to reduce the number of hops and to improve latency, as shown in Figure 9. This characteristic calls for topology management combined with dynamic channel selection to select the best routes. Ref [14] addresses the problem of topology management by proposing a backbone architecture with cluster heads and gateway nodes that manage capacity consumption. The authors implemented a reinforcement learning-based joint dynamic channel selection and topology management method. In their architecture, sensing is achieved by deploying specialized spectrum sensing devices called coordinators. However, the nodes along this backbone will always be put into use, which will, in the long term, jeopardize the existence of the entire network. The authors attempted to solve this problem by assuming the presence of high-energy nodes along the backbone route. This assumption, however, is not always practical. In contrast, [49] proposes a multi-layered architecture for CRWSNs to provide energy and spectrum efficiency for smart grid utilities. The main point derived here is that in designing routing protocols, the protocol should have the ability to adequately address the topology management challenge, including tackling the deafness, hidden and exposed terminal challenges.

#### 9.1.2. Route Setup

The route setup scheme is also application-specific. Generally, its classification is similar to the case of a CRAHN, as presented in Section 7 above. The main issues that the route setup must tackle include spectrum sensing, control signaling and channel decisions.

##### 9.1.2.1. Spectrum Sensing

Spectrum sensing is the process whereby the nodes obtain spectral awareness of their environment in terms of free channels and the behavior of the PU on those channels. It is important to note that sensing is not restricted to discovering spectrum opportunities. Sensing is also used to detect the presence of incumbent users and to vacate the spectrum to avoid interfering with PU communication. Because the nodes we are considering have only single transceivers, the activity of both sending and sensing becomes very challenging, unlike in the case of multiple transceivers wherein the duties can be shared between the various radio interfaces [50]. However, the fact that the power of sensor transmission rarely affects the transmission of a PU downplays the issue somewhat. In the case of sensing for spectrum awareness, we can base the route setup on full spectrum awareness or local spectrum awareness scenarios.

###### 9.1.2.1.1. Full Spectrum Awareness

For the full spectrum scheme, knowledge of the spectrum availability and channel characteristics is already available and properly documented in specialized servers. Thus, SUs only need to have access to the servers that host the database and can then make communication schedules based on the information. The idea of having a centrally maintained spectrum database that will indicate channel availabilities in the spectrum below 900 MHz and at approximately 3 GHz over time and space is being promoted by the FCC [51]. In this light, [52,53] proposed measurement-based sensing and modeling techniques for categorizing the channel availability to ease the process of sensing. For resource-constrained nodes, this scheme will be most favorable for enhancing route setup because it solves the problem of trying to synchronize the quiet periods of nodes for spectrum sensing. This approach also gives nodes more time for data transmission because it is known that quiet periods actually reduce node transmission periods. In the long run, this scheme makes route scheduling very effective and can effectively manage mobile nodes.

###### 9.1.2.1.2. Local Spectrum Awareness

For the local spectrum scenario, nodes must build the spectrum occupancy database based on their local sensing activity. For sensor nodes, it will be impossible for each sensor to sense through the whole spectrum for opportunities; thus, adequate sensing schemes that are suitable for sensor nodes and can meet service demands must be implemented. Ref [54] advocates the need for spectrum sensing algorithms that utilize a minimum number of samples to detect PUs within a specific detection error probability to improve energy conservation/minimization of transmitting nodes.

In this respect, cooperative sensing schemes are more favored over individual sensing because distributing the duty of spectrum sensing among the nodes can drastically reduce the energy demand on the nodes and increase the time for data transfer. In this light, [55] advocates that cooperative sensing is more favorable for request to send (rts) effectiveness in improving SU detection accuracy, but the issues of information fusion and distributed spectrum sensing are still open.

Ref [56] proposes a method of cooperative spectrum management. This two-layered hierarchical model uses a centralized sensing scheme. However, their proposal does not practically address the main issues of CR such as DSA, spectrum handoff, *etc.* Other credible concepts that can be utilized or incorporated while designing distributed sensing algorithms have been presented in [57], which minimizes the energy used in distributed sensing by obtaining a Negmon-Pearson and Bayesian formulation to optimally choose the sleeping and censoring design parameters, and [58], which develops distributed spectrum sensing and channel selection for WPANs. The learning engine derives its cognition from the exchange of SOP. Various learning schemes have been implemented in this regard to help the cognitive engine gradually build up a knowledge base of spectrum opportunities so that it can quickly adapt to its environment to make better, more informed routing decisions. This method is similar to that presented in [59], which is a DSA scheme with learning for CR conducted by awarding weights based on the learning experience.

##### 9.1.2.2. Control Signaling

Control signaling is the process whereby nodes with data to send to the sink negotiate for a path with their neighbors via a CCC. Although Section 3.2.1 laid the basis for control signaling, we only mention the most relevant studies in this respect under the following categories: dedicated common control channel, sequence-based control channel negotiation and group-based control channel.

###### 9.1.2.2.1. Dedicated Common Control Channel

Several studies [60] that use a dedicated common control channel [61,62] usually assume its availability without providing details about how such a channel is secured. This fact alone indicates the need for securing the design of such a channel. Again, the most relevant work in this respect is [63], which proposed the design of an out-of-band CCC using the guard bands between the channels of the licensed spectrum. This design was achieved by carefully selecting relevant subcarrier parameters such as transmit power, bandwidth and the maximum possible number based on the constraints of OFDM technology and the permissible levels of spectral overlap with the PU transmission. In this scheme, the CR nodes are also able to selectively activate guard bands based on the local observed PU activity. However, the implementation of OFDM technology that was originally developed for wideband digital communication on the platform of single-transceiver CRSN communication is still an open issue.

###### 9.1.2.2.2. Sequence-Based Control Channel Negotiation

For sequence-based schemes, in [64], CR nodes broadcast their available channels in all of the licensed channels. Time in this case is divided among the same number of available channels, with each channel being assigned a slot at the beginning of the network. Any neighbor having channels in common with the broadcasting neighbor also updates its list. For data transfer, a CR node must wait for the slot corresponding to the channel it has in common with its neighbors. Thus, CR nodes hop along a channel sequence that can differ from that of its neighbor transmitting packets, indicating that it has data to transfer. Once a node pair exchanges the synchronization packets on a common channel, they then decide on a common hopping sequence for the data transfer. The overhead incurred for broadcast and the time needed to establish a link at every instance of data transfer make this scheme too costly for CRSNs. For [20,65], the process of weighted hopping to secure the control channel is only performed once backup channels are established in case the first one is lost due to extended PU activity. In contrast, [64] proposes an alternative MAC protocol that does not require a common channel for multi-hop CR networks. The time is divided into fixed slots, and all users listen to a channel at the beginning of each slot.

###### 9.1.2.2.3. Group-Based Control Channel

The fact that group (*i.e.*, cluster)-based algorithms are best suited for establishing sub-network-wide or complete network-wide coverage of the chosen control channel makes it the method of choice for CRSNs. This trend is due to a number of reasons. Ref [59] showed that due to spatial correlation availability, there is a high possibility of finding a common channel in certain restricted areas. The issue of congestion of the CCC has been downplayed by the 15%–22% CCC usage, as illustrated in [44], and its potential of enhancing throughput [66].

The most relevant work in this respect is [67] in which dedicated control channels are assigned by cluster heads to members based on the sensing results of the cluster members. As in traditional clustering algorithms, the manner in which the deafness issue is overcome is not mentioned. Thus, the probability of potential cluster members receiving the broadcast of neighbors cannot be ascertained. This scenario can best be described as a blind rendezvous scheme in which a rendezvous cannot be assured unless it is methodically planned, especially in the presence of multiple cluster members. Another major feature of the protocol is the decision scheme for selecting the control channel based on an approximated partially observable Markov decision process (A-POMDP) and the channel availability (CA) of both the PUs and SUs.

Another significant work in this respect for traditional CRAHNs is [68], which proposes a design approach for an efficient recovery control channel (ERCC) that consists of three components: neighbor discovery, control channel (CCL) update and efficient PU activity recovery. Again, the traditional approach for cluster formation was followed. However, the issue of blind rendezvous was properly addressed. In the ERCC, the cluster formation is based on a rigorous neighborhood discovery scheme, while [20,65] make this process very simple by introducing the concept of virtual clustering, which maximizes the utilization of idle listening.

##### 9.1.2.3. Channel Decision

In a bid to initiate the routes, SOPs must be characterized based on the different preferences of either of the following: complete avoidance of PU zones, the FCC interference temperature standard limit [69] or prediction schemes. Relevant studies in these regards are presented below.

###### 9.1.2.3.1. Complete Avoidance of PU Zones

This option is selected to reduce or eliminate spectrum handoff. The resulting implication is that the chosen route might happen to be the longest path to the sink. One study based on this scheme is [70], where the authors computationally analyzed the algorithm to guarantee fair spectrum allocation while minimizing handoff. Because spectrum handoff consumes approximately 96.0% of the average receiving energy or 110.75% of the average transmit energy, the authors attempted to minimize handoffs (*i.e.*, limiting unnecessary spectrum handoffs). They used a modified game theory (MGT) strategy to fairly allocate spectrum bands (based on sensor weights, *i.e.*, prioritizing the nodes for fairness) while avoiding bands that have a high probability of PU activity to minimize handoffs. The authors formulate the centralized spectrum allocation problem into a multi-objective nonlinear programming problem and then solved it with MGT. However, they did not take congestion into consideration, and their solution can be said to be computationally complex.

In [61] channels with the least probability of PU activity to nodes in a cluster-based network are assigned. They examine three channel assignment approaches (random-pairing, greedy-pairing and optimization-based channel assignment schemes) while basing the last two on their R-coefficient metric (a dual metric that represents the residual energy of the nodes and the channel conditions). Their priority was to extend both node and network lifetime. The authors also made an assumption about the availability of a control channel that is used by the CH to allocate channels among cluster members. [71] proposes a frequency-hopping algorithm to reduce the interference time *in lieu* of the QP algorithm. The authors pre-allocate frequency channels for each hopping period at the beginning of the data transmission period using a prediction algorithm that utilizes post-spectrum sensing information. Their channel utilization algorithm is based on a hidden Markov model (HMM). Their main aim was to increase throughput. However, the idea of implementing the presented HMM using resource-constrained nodes can also be said to be computationally demanding and complex. According to [72], a satisfactory real-time constant bit rate (CBR) and best effort (BE) can both be guaranteed in the presence of periodic frequency switching (PS) and triggered switching (TS). TS exhibited better throughput than PS. Ref [73] also proposes a cooperative energy PU detection method. The authors consider the practicability of their proposal by studying the performance when quantization is applied to the energy values before transmission. Their results showed a performance comparable to that of the optimum N-P test at 4–quantization, with the advantage of having less overhead.

###### 9.1.2.3.2. FCC Interference Temperature Limit

The idea behind this scheme is that SUs are allowed to transmit in the same spectrum in the presence of PUs as long as they do not exceed a set interference temperature level above the noise floor. Although the interference temperature limit concept was terminated in 2007 by the FCC [74] based on a lack of specific implementation rules, research to define these rules is ongoing because of its suitability in the realms of CRSNs. This scheme requires the SU to know the location of the PU, which necessitates polynomial calculations for precise interference measurement. An example of a study based on this model is [75], which models the interference temperature dynamics of a primary channel with a Baum-Welch trained HMM that they proved to be statistically stable. SUs use the trained HMM to predict the channel's interference temperature in future time slots and calculate the channel availability metric value for the channel in question. This value is, in turn, used by the SU underlay for primary channel selection and transmission. Ref [76] presents a mathematical model that serves as an interference avoiding mechanism for aggregated interference to the primary networks. In a bid to explore the possibilities of CRSNs amidst various interferences, [9] proposes a model that determines the optimum transmit power required to achieve a desired throughput by measuring the interference temperature. However, various studies have consistently shown the limitations of this method. Additionally, a partially observable Markov decision process (POMDP) framework for a decentralized cognitive MAC for OSA has also been proposed [77]. The scheme properly resolves the hidden and exposed terminal issues and exploits opportunities at the slot level. The results showed optimized SU performance while limiting the interference perceived by the PUs.

###### 9.1.2.3.3. Prediction Schemes

Prediction schemes are usually used to predict the arrival of PUs so that effective actions will be taken in a timely manner to avoid data loss or time-outs. In this respect, [78] introduces a channel-aware transmission mechanism based on CSMA to optimize energy efficiency. The pushback mechanism simply predicts the channel status to determine the optimal times to transmit and to refrain from transmission to conserve energy. Without additional packet overhead and with its minimal computational requirements, the mechanism significantly improves the packet success rate and the energy without degrading throughput. This result was achieved by modeling the channels as an HMM. Another related work along this line is [79], which presents a MAC layer spectrum sensing scheme using random sampling and sensing based on a maximum likelihood (ML) estimation strategy. The authors focused on both scheduling the sensing activity and the estimation of statistical properties of random variations in the channel.

#### 9.1.3. Route Management

After the preferred route is chosen, it must be maintained throughout the period of data transfer from hop to hop. This module must be attentive in case of a sudden PU arrival and should have adequate ways of seamlessly handing the link to the next spectrum or channel, as the case may be. At a glance, the possibilities of frequent interruption during the data transfer phase usually make the idea of accommodating CBR traffic in CR schemes appear vague. However, [80] shows the possibility of guaranteed real-time CBR traffic over a CRSN. Additionally, [72] shows that satisfactory real-time CBR and BE can both be guaranteed in the presence of PS and TS, although TS exhibited a better throughput than PS. However, the route maintenance module should be able to discover new paths in case of the failure of already established paths, especially when mobility is considered.

Other factors to consider during route maintenance include path delay, energy and path reliability. Any protocol should incorporate these metrics to maximize network resources because route maintenance only occurs during data transfer, which is the most resource-demanding phase. In this respect, [81] presents a spectrum-aware routing scheme for CRSNs by estimating the spectrum usage of both PUs and SUs using Bayesian learning. The metrics used were reliability, energy consumption and path delay. These metrics were combined using a multiple attribute decision making (MADM) algorithm. Their results showed a better reliability than the scenarios strictly based on power and delay metrics alone. Although the report claimed better performance in power management with respect to WSN scenarios, this finding was not illustrated. The performance was also not compared to established WSN and CRAHN solutions. Hence, the claims to adequacy cannot be verified.

## 10. Conclusions/Outlook

In this work, we have further justified the CRSN paradigm as a NextGen solution and have also attempted to identify a research gap in the network layer. From our analysis, we have demonstrated the insufficiency of presenting routing solutions from WSNs to properly fill this gap. We have shown that to properly handle the dynamic environment, a protocol should integrate proactive and reactive components and should have a controlled redundancy of data packets, depending on the nature of the route selected. A detailed discussion of the pros and cons of various techniques has been presented for the critical steps of routing in this regard. We have reaffirmed the imperative of taking the cross-layer approach and have developed a framework to that effect; finally, we have also presented a draft framework for routing in CRSNs. Future work includes the design of a full network management and routing protocol that takes the aforementioned issues into consideration.

## Figures and Tables

**Table 1.** Simulation parameters.

**Parameters**	**Values**
Distance (X,Y)	(1,1)
Number of nodes	50
Application source, center type, radius, rate, random rate	Static, random, 1, 4, 0
Application destination type, center type, radius, rate, random rate	Static, random, 1, 0.5, 0
Packets generated	Infinity
Data traffic	Constant bit rate (CBR)
Data rate	250 kbps
Initial node energy	30 Joules
Simulation time	100 s
Routing protocol	RTLD, AODV, MCBR, SC, FF, FP
Window size, C1, Z	10, 0.7, 1
MCBR learning rate, Resend, ForwardDelta, MaxDelay, Flood Temp	1, 1, infinity, 4,000, 5
AntStart, Ratio, RewardScale, DataGain	120,000, 2, 0.3, 1.2
Transmission Timeout, Retries, MaxHops	3500, 8, Infinity
AODV RQcache, Rtable, RREP Retries, RREP delay, RREQ Timeout	10, 10, 3, 60,000, 400,000
